# A Rare Case of a Failed AngioVac Procedure Used to Debride Tricuspid Vegetation Complicated by Ogilvie Syndrome

**DOI:** 10.7759/cureus.23584

**Published:** 2022-03-28

**Authors:** Rafail Beshai, Howard Weinberg

**Affiliations:** 1 Internal Medicine, Jefferson Health New Jersey, Stratford, USA; 2 Cardiology, Virtua, Camden, USA

**Keywords:** angiovac, ogilvie syndrome, ogilvie's syndrome, complication, tricuspid vegetation

## Abstract

Ogilvie syndrome is a rare disorder characterized by dilatation of part or all of the colon and rectum without intrinsic or extrinsic mechanical obstruction. Its etiology is likely multifactorial with high mortality if left untreated. Here, we report for the first time a case of Ogilvie syndrome secondary to the AngioVac procedure. Because our patient had a high operative risk, we used the AngioVac system to debulk tricuspid valve vegetations to reduce bacterial load. Although AngioVac is considered safe overall, publications describing its side effects, safety, and efficacy are limited. Providers should be aware of this rare but potentially fatal complication and the importance of close clinical monitoring and serial abdominal examinations following AngioVac procedures.

## Introduction

The number of infective endocarditis (IE) cases has been increasing recently. In particular, the proportion of patients with prosthetic valve endocarditis has increased [1]. Although new diagnostic and treatment strategies have emerged, one-year mortality has not improved and remains at 30% [2]. Right‐sided IE accounts for only 5% of all IE cases [3]. Compared with the extensive data on left‐sided IE, there is much less published information regarding the features and management of right‐sided IE [4].

AngioVac was approved in 2014 for the removal of fresh, soft thrombi or emboli. However, in recent years, several case reports and case series have introduced the AngioVac system to debulk tricuspid valve vegetations in patients with a high operative risk [5]. This concept of debulking tricuspid valve vegetations aims to reduce bacterial load to allow antimicrobial therapy to cure the infection. Given that AngioVac was recently approved by the Food and Drug Administration (FDA) and due to a limited number of case reports, publications about its side effects, safety, and efficacy are limited. To our knowledge, after performing an extensive literature review on PubMed and Google Scholar, we believe that this is the first case describing Ogilivie syndrome secondary to AngioVac. Clinicians should be aware of this unique side effect to aid in proper monitoring, treatment, and management.

## Case presentation

A 31-year-old female known to be an intravenous (IV) drug user presented to the hospital with shortness of breath, fever, and atypical chest pain. Although the episode had started a week before the presentation, it was progressively worsening. She stated that she felt similar to when she had needed a tricuspid valve replacement the previous year. Her vital signs upon arrival were as follows: temperature 38.2°C, heart rate 110 beats per minute, blood pressure 130/80 mmHg, and oxygen saturation 97% on 4 L of nasal cannula. Her cardiac examination was notable for a tricuspid regurgitation murmur, regular rhythm, and normal S1 and S2.

Her medical history was significant for tricuspid valve replacement for endocarditis, pericardial window complicated by right ventricular perforation, current IV drug abuse (heroin and fentanyl), and septic pulmonary emboli. She had ongoing bacteremia status post new peripherally inserted central catheter (PICC) and had been on numerous antibiotics/antifungals, including piperacillin/tazobactam and Synercid. She was previously on micafungin for over six weeks. She had also completed a course of daptomycin/rifampin and was on Levaquin upon admission.

Given the patient’s chest pain, an electrocardiogram (EKG) was done, and troponins were drawn. Her EKG showed sinus tachycardia with an otherwise normal EKG. Her high-sensitivity troponin was negative X3. An X-ray showed small left pleural effusion and left lower lung atelectasis and/or infiltrate. CT angiogram was negative for pulmonary embolism. Given the patient’s ongoing bacteremia, fever, and IV drug abuse, a transthoracic echocardiogram was done which showed large vegetation on a bioprosthetic tricuspid valve (Video 1). The mean gradient was severely elevated (14 mmHg) across the tricuspid valve, likely due to obstruction from leaflet vegetation. It also showed moderate tricuspid regurgitation. Her blood cultures showed polymicrobial bacteremia of methicillin-resistant *Staphylococcus aureus*, *Candida glabrata*, *Escherichia coli*, *Klebsiella*, vancomycin-resistant *Enterococcus*, and *Pseudomonas* due to IV drug abuse. The patient’s basal metabolic panel showed that she had an acute kidney injury.

**Video 1:** Long-axis right ventricular inflow tract view showing large vegetation on a bioprosthetic tricuspid valve. Blue arrow showing large vegetation on a bioprosthetic tricuspid valve.

Given that she had bacteremia and acute kidney injury, she was not a surgical candidate, and a decision to pursue AngioVac was made. The patient was brought to the hybrid operating room. Access to the bilateral common femoral veins was obtained. Venovenous bypass was commenced and multiple attempts at debriding the prosthetic tricuspid valve were performed without success. A transesophageal echocardiogram revealed that most of the vegetation burden was on the ventricular side of the valve. Venovenous bypass was commenced once again. Another attempt to debride the valve from the ventricular side was started. Multiple passes were performed. The mass and vegetation did appear to be engaged by the AngioVac device, but it was very resistant to debridement (Video 2). After multiple passes without success and with the patient starting to have a run of ventricular tachycardia, the procedure was stopped.

**Video 2:** The AngioVac cannula (shown by a blue arrow) inserted through the femoral vein under X-ray guidance in an attempt to drain the target vegetation under TEE guidance (red arrow). TEE: transesophageal echocardiogram

**Figure 1:**
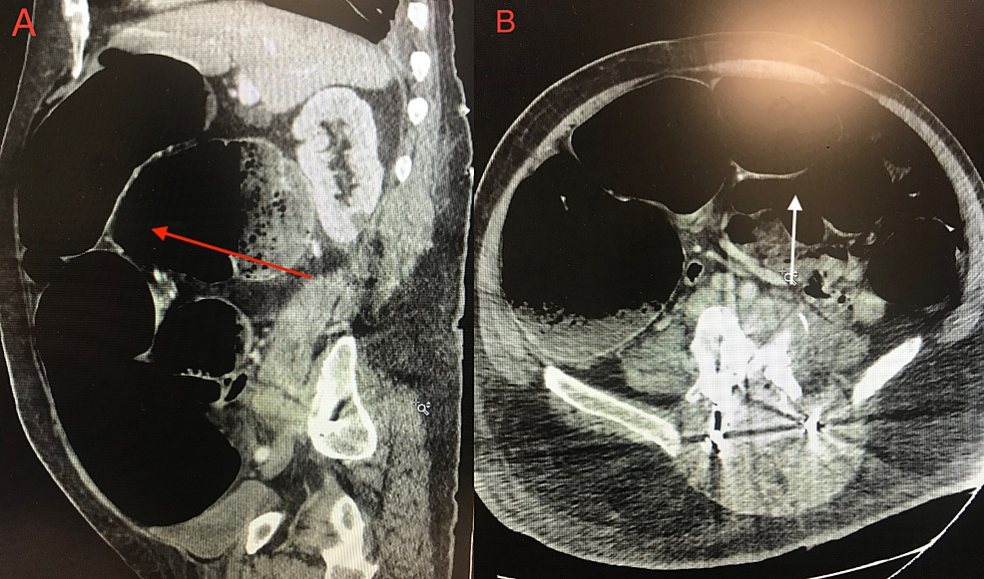
(A) Sagittal view of CT abdomen showing acute colonic dilation shown by the red arrow. (B) Coronal view of CT abdomen showing acute colonic dilation shown by the white arrow.

**Figure 2:**
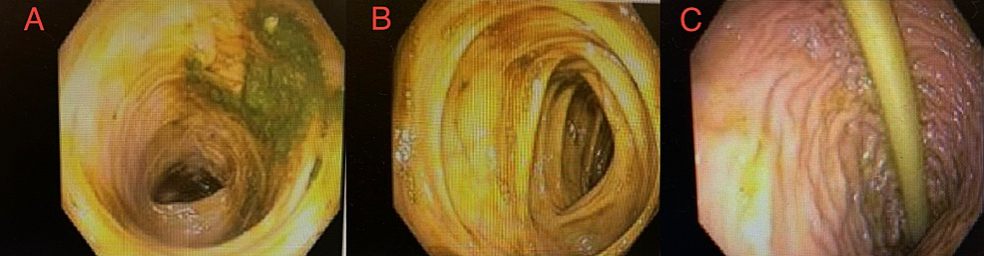
(A) Flexible sigmoidoscopy showing descending colon without mechanical obstruction. (B) Flexible sigmoidoscopy showing transverse colon without mechanical obstruction. (C) Flexible sigmoidoscopy showing rectal tube is in place.

The patient started developing rapidly progressive abdominal distension with nausea and vomiting in the next 24 hours following the AngioVac procedure. CT of the abdomen showed acute colonic pseudo-obstruction, which is also known as Ogilvie syndrome (Figures 1A, 1B). The patient was advised to withhold oral food and fluid intake. She was started on IV normal saline. A nasogastric tube was placed. Unfortunately, because the patient did not improve in the next 48 hours, a decision was made to perform decompressive flexible sigmoidoscopy with rectal tube placement (Figures 2A-2C).

Over the next 48 hours, the patient felt better while passing flatus and bowel movements. Her diet was advanced, and she was able to tolerate regular food. Regarding the tricuspid vegetation, the patient was discharged on long-term antibiotics using her PICC line. A repeat transthoracic echocardiogram done a month later showed a decrease in the vegetation size with significant improvement in her symptoms. She was advised to seek help regarding her drug addiction, and she showed understanding and agreed to go to a drug rehab facility.

## Discussion

Ogilvie syndrome, also known as acute colonic pseudo-obstruction, is a rare disorder characterized by dilatation of a part or all of the colon and rectum without intrinsic or extrinsic mechanical obstruction [6]. It was first described in 1948. The exact incidence of Ogilvie syndrome is unknown. Although its pathophysiology remains poorly understood, it appears to be multifactorial [7]. The etiology is most likely a combination of dysregulation of the autonomic nervous system, decreased splanchnic perfusion, and decreased prostaglandin [7-9].

Oglivie syndrome can be life-threatening if left untreated with high mortality rates [2]. Studies have shown Ogilvie syndrome as a postoperative complication occurring most commonly after major obstetrical/gynecologic, abdominal/pelvic, orthopedic, and coronary artery bypass procedures [10,11]. However, some case reports have shown that it may occur after small easy procedures such as cardioversion for atrial fibrillation [12].

Diagnosis is suggested by a physical examination and CT scan which shows dilation with no obstructive lesions or bowel ischemia. Ogilvie syndrome is treated based on the 2010 American Society of Gastrointestinal Endoscopy guidelines [13]. Conservative management should be tried first by putting the patient nil per os (NPO) and insertion of a nasogastric tube and IV fluid with electrolyte repletion. Neostigmine is the next step on the ladder; however, given our patient’s history of previous cardiac arrest, a decision was made to pursue endoscopic colonic decompression. The last step is surgery; fortunately, our patient did not need to undergo surgery.

## Conclusions

Ogilvie syndrome is a rare but potentially fatal complication following the AngioVac procedure. This case acts as a reminder of the importance of close clinical monitoring and serial abdominal examinations following the AngioVac procedure.
